# Pattern of cortical thinning in logopenic progressive aphasia patients in Thailand

**DOI:** 10.1186/s12883-020-02039-x

**Published:** 2021-01-13

**Authors:** Sekh Thanprasertsuk, Yuttachai Likitjaroen

**Affiliations:** 1 Faculty of Medicine, Chulalongkorn University, 1873 Rama 4 Road, Patumwan, Bangkok, 10330 Thailand; 2 Chulalongkorn Cognitive Clinical & Computational Neuroscience Special Task Force Research Group, Chulalongkorn University, Bangkok, Thailand; 3 Chula Neuroscience Center, King Chulalongkorn Memorial Hospital, The Thai Red Cross Society, Bangkok, Thailand

**Keywords:** Aphasia, primary progressive, Logopenic progressive aphasia, Magnetic resonance imaging, Alzheimer’s disease, Freesurfer, Cortical thickness

## Abstract

**Background:**

Logopenic progressive aphasia (LPA) is an uncommon neurodegenerative disorder primarily characterized by word-finding difficulties and sentence repetition impairment. Prominent cortical atrophy around left temporo-parietal junction (TPJ) is a classical imaging feature of LPA. This study investigated cortical thinning pattern in clinically diagnosed LPA patients using non-demented subjects as a control group. We also aimed to explore whether there was prominent thinning of other cortical area additional to the well-recognized left TPJ.

**Methods:**

Thicknesses of all cortical regions were measured from brain magnetic resonance images using an automated command on *Freesurfer software.* Cortical thickness of the LPA and control groups were compared by two methods: 1) using a general linear model (GLM) in *SPSS software*; and 2) using a vertex-by-vertex GLM, performed with *Freesurfer’s QDEC interface.*

**Results:**

Besides the well-recognized left TPJ, cortical regions that were significantly thinner in the LPA group by both comparison methods included left caudal middle frontal gyrus (CMFG) (*p* = 0.006 by *SPSS*, *p* = 0.0003 by *QDEC*), left rostral middle frontal gyrus (*p* = 0.001 by *SPSS*, *p* = 0.0001 by *QDEC*), left parahippocampal gyrus (*p* = 0.008 by *SPSS*, *p* = 0.005 by *QDEC*) and right CMFG (*p* = 0.005 by *SPSS*, *p* = 0.0001 by *QDEC*).

**Conclusions:**

Our results demonstrated that thinning of middle frontal gyri may be an additional feature in clinically diagnosed LPA patients. Involvement of left parahippocampal gyrus may reflect the underlying neuropathology of Alzheimer’s disease in majority of the LPA patients.

**Supplementary Information:**

The online version contains supplementary material available at 10.1186/s12883-020-02039-x.

## Background

Logopenic progressive aphasia (LPA) is a neurodegenerative disorder which has been classified as a logopenic variant of primary progressive aphasia (lvPPA, PPA-L) [1–3]. Alzheimer’s disease (AD) pathology was found to be an underlying neuropathology in a majority of LPA patients in autopsy studies [4, 5]. Amyloid-β burden, which strongly related to AD pathology, was also found in more than 80% of LPA patients by imaging and cerebrospinal fluid analyses [4]. LPA patients normally presented with predominant language abnormalities including word-finding difficulties, anomia, verbal working memory impairment and deterioration of sentence repetition, while episodic memory is initially intact [1–3]. Therefore, LPA has also been considered as a variant of AD since it is often underlaid by AD pathology, but the manifestation is not the typical amnestic presentation of AD in the early stage [1].

A neuropathological study revealed that LPA patients had predominated pathology around the temporoparietal junction (TPJ) including superior temporal gyrus (STG) and middle temporal gyrus (MTG) of the left cerebral hemisphere [5]. The findings were in accordance with several cortical thickness measurement studies [6–9]. This anatomical pattern of cortical thinning corresponded well with the clinical manifestations since left TPJ had important roles on word retrieval, verbal working memory and speech repetition [3, 10, 11]. Some LPA patients, however, had nearly normal naming ability despite markedly poor verbal fluency, suggesting greater impairment of word retrieval elicited by internal language substrates than by external perception [3]. Thus, alternative cortical language area involving in different steps of word selection might be deteriorated in these LPA patients.

This is the first study of cortical thinning pattern in LPA patients in Thailand. We aimed to investigate pattern of cortical thinning in LPA patients to explore whether there was prominent thinning of other cortical area additional to the well-recognized left TPJ, by using non-demented subjects as a control group. Thicknesses of all cortical regions were measured from brain magnetic resonance images (MRI) using an automated command on *Freesurfer software.* Cortical thickness of the two groups were then compared by two methods: 1) using a general linear model (GLM) in *SPSS software*; and 2) using a vertex-by-vertex GLM, performed with *Freesurfer’s QDEC interface.*

## Methods

### Study population

This is a retrospective study. LPA patients were recruited from the Neurocognitive Clinic, Division of Neurology, Department of Medicine, King Chulalongkorn Memorial Hospital, Bangkok, Thailand. They were diagnosed as LPA according to the diagnostic criteria [2]. Control subjects were patients who had been consulted to the Neurocognitive Clinic subjectively complaining of working memory problem without impairment of other cognitive domains. They also had normal Thai Mental State Examination (TMSE) test result [12], i.e. TMSE score of more than 23, and had normal brain MRI findings. We could thus presume that all the control subjects were not demented and had no objective clinical evidence of neurodegenerative disorder at the time we recruited. LPA patients and control subjects had completed brain MRI data during January 2014 to July 2018. Participants were excluded if (1) they had history of disease contributing to brain atrophy other than neurodegenerative disorder including cerebrovascular disease, chronic inflammatory disorder, cancer, chronic liver disease, severe head injury, encephalitis and toxic encephalopathy; (2) there were large structural abnormalities on brain MRI preventing an accurate assessment of cortical thickness or brain volume; or (3) they had brain MRI of insufficient quality. All participants signed a written informed consent for using the patient and MRI data prior to the enrollment in the study. General information of the participants including age, sex, years of education and TMSE score on the first visit date at the Clinic were recorded. In this study, age was counted on the date MRI performed.

### Brain MRI

Brain MRI was routinely obtained for diagnosis within 2 months after the patient first visit at the Neurocognitive Clinic. The protocol used in this study included high resolution 3-dimension T_1_-weighted imaging for structural study and cortical thickness measurement, and fluid attenuated inversion recovery (FLAIR) T_2_-weighted imaging for visualizing pathologic lesions.

### Imaging acquisition

The scanner was 3 Tesla brain MRI scanner of Philips Medical Systems, Best, The Netherlands (MR System Ingenia, software release 5.1). The acquisition for T_1_-weighted image was isometric with sensitivity encoding, sagittal T_1_-weighted 3-dimensional turbo field echo, repetition time (TR)/echo time (TE) = 8.1 ms/3.7 ms, flip angle 8 degrees, voxel size 1.00 × 1.00 × 1.00 mm^3^ 160 slices without gap. The acquisition for FLAIR image was axial plane TR/TE = 11,000 ms/125 ms, TI = 2800 ms, voxel size = 0.7 × 1.60 × 6.0 mm^3^, 20 slices 6 mm slice thickness with gap = 1 mm.

### Brain image processing for cortical thickness measurement and use of post-processing data

The image processing was performed using *FreeSurfer-i386-apple-darwin11.4.2-stable5–20,130,514* which is freely-available (http://surfer.nmr.mgh.harvard.edu/). The detailed procedure for the measurement has been described and validated in previous studies [13–15]. Briefly, pre-processing of all patients’ images was done by entering automate *“recon-all”* command to perform Talairach registration, intensity normalization, skull stripping, brain segmentation, tessellation of the grey and white matter boundary, topology correction, and cortical surface reconstruction and parcellations. Finally, before group comparison process, the reconstructed cortical surface was smoothed with a 10 mm full-width at half maximum surface-based Gaussian kernel to reduce local variation in the measurements. This processing method was shown to be reliable in a series of healthy elderly subjects [16]. Post-processing data including total brain volume (cm^3^), overall averaged cortical thickness (mm) and cortical thickness of each brain region (mm) were used for statistical comparison between groups.

### Statistical analysis

Comparisons of demographic and clinical characteristics between LPA and control groups were performed by appropriate independent sample tests in *SPSS Statistics Version 23.0* (IBM Corp., Armonk, NY). Age and total brain volume were compared by using unpaired t-test. Sex proportion was compared by using Fisher’s exact test. Years of education and TMSE score were compared by using Mann-Whitney U test. Relationship between cortical thickness and participant characteristics were analysed by appropriate statistics: Pearson’s correlation for age; Spearman’s correlation (ρ) for years of education and TMSE score; and unpaired t-test for sex. Cortical thickness was compared between the LPA and control groups using two methods. Firstly, we compared the average thickness of each parcellated cortical area between the two groups using *SPSS software*. A multivariate general linear model (GLM) controlling for age, sex and total brain volume was applied for this comparison. The *p-value* was set at 0.01. Secondly, we adapted a previously described vertex-by-vertex GLM method performed with *Freesurfer’s Query, Design, Estimate, Contrast (QDEC) Interface* [11, 17]. In this second method, pial surface templates of left and right hemispheres, which allow visualization of data across the entire cortical surface, were generated. Clusters showing significant difference of averaged cortical thickness between the two groups were then overlaid on the brain templates as highlighting areas. The analyses in *QDEC* were corrected for multiple testing using Monte Carlo simulations with *p*-value ≤0.01 [18].

## Results

### Characteristics of LPA and control groups

There were 10 LPA patients and 20 control subjects recruited in this study (Table 1). LPA patients were significantly younger than control subjects (69.3 ± 6.7 vs 76.1 ± 6.4 years old, *p* = 0.02) and majority of them were male (7 patients [70%] vs 3 patients [15%], *p* = 0.003). All participants were right-handedness. Years of education was not different between LPA and control groups (*p* = 0.98). TMSE score was significantly lower in the LPA group (*p* < 0.001). Total brain volume was not significantly different between the two groups. Despite the comparable brain volume, overall cortical thicknesses of both left and right hemisphere were significantly thinner in LPA patients than in control subjects (left hemisphere: 2.20 ± 0.17 vs 2.41 ± 0.10 mm, *p* = 0.003; right hemisphere: 2.24 ± 0.17 vs 2.42 ± 0.10 mm, *p* = 0.008). All participants from both groups had Fazekas scale 1 for white matter hyperintensities visualized in FLAIR images.

**Table 1:** Characteristics of LPA and control groups

Characteristics	LPA (***n*** = 10)	Control (***n*** = 20)	***p***-value
Age, years, mean (SD)	69.3 (6.7)	76.1 (6.4)	0.02
Sex, female, *n* (%)	3 (30.0%)	17 (85.0%)	0.003
Education, years, median (IQR)	16.0 (4.0)	16.0 (5.5)	0.98
TMSE score, median (IQR)			
Language section (max. 10)	7.0 (1.0)	9.0 (1.0)	< 0.001
Non-language sections (max. 20)	12.5 (2.5)	19.0 (1.8)	< 0.001
Total (max. 30)	19.5 (3.5)	28.0 (1.0)	< 0.001
Brain volume, cm^3^, mean (SD)	1009.23 (155.46)	956.16 (105.19)	0.35
Overall cortical thickness, mm, mean (SD)			
Left hemisphere	2.20 (0.17)	2.41 (0.10)	0.003
Right hemisphere	2.24 (0.17)	2.42 (0.10)	0.008

Regarding the clinical information of participants in the LPA group, the average age of the symptom onset was 63.4 ± 4.79, ranging from 55 to 70 years old. All of them had striking impairment of single-word retrieval and sentence repetition, while fluency of spontaneous speech was relatively spared. They had neither word comprehension failure nor frank agrammatism. First structural brain MRI demonstrated prominent brain atrophy around left TPJ area in 6 (60%) patients. For the remaining 4 LPA patients, such imaging characteristic was more obviously visualized on the follow-up structural brain MRI a year later. Based on the diagnostic criteria, all 10 patients were thus consistent with the “imaging-supported LPA diagnosis” [2].

For the relationship between overall cortical thickness and participant characteristics, the total TMSE score was found to be positively correlated with overall cortical thickness in both left (*ρ* = 0.70, *p* < 0.001) and right hemispheres (*ρ* = 0.62, *p* < 0.001). Score from the language section of TMSE also had positive correlations with overall cortical thickness on both left (*ρ* = 0.58, *p* = 0.001) and right hemispheres (*ρ* = 0.51, *p* = 0.004). Relationship analyses between TMSE score and thickness of each parcellated cortical region additionally showed significant positive correlation in most areas (Supplementary Table 1). While other participant characteristics including age, sex and educational years showed no relationship with overall cortical thickness in both hemispheres (*p* > 0.05).

### Comparing cortical thickness between groups using GLM in SPSS software

**Fig. 1:**
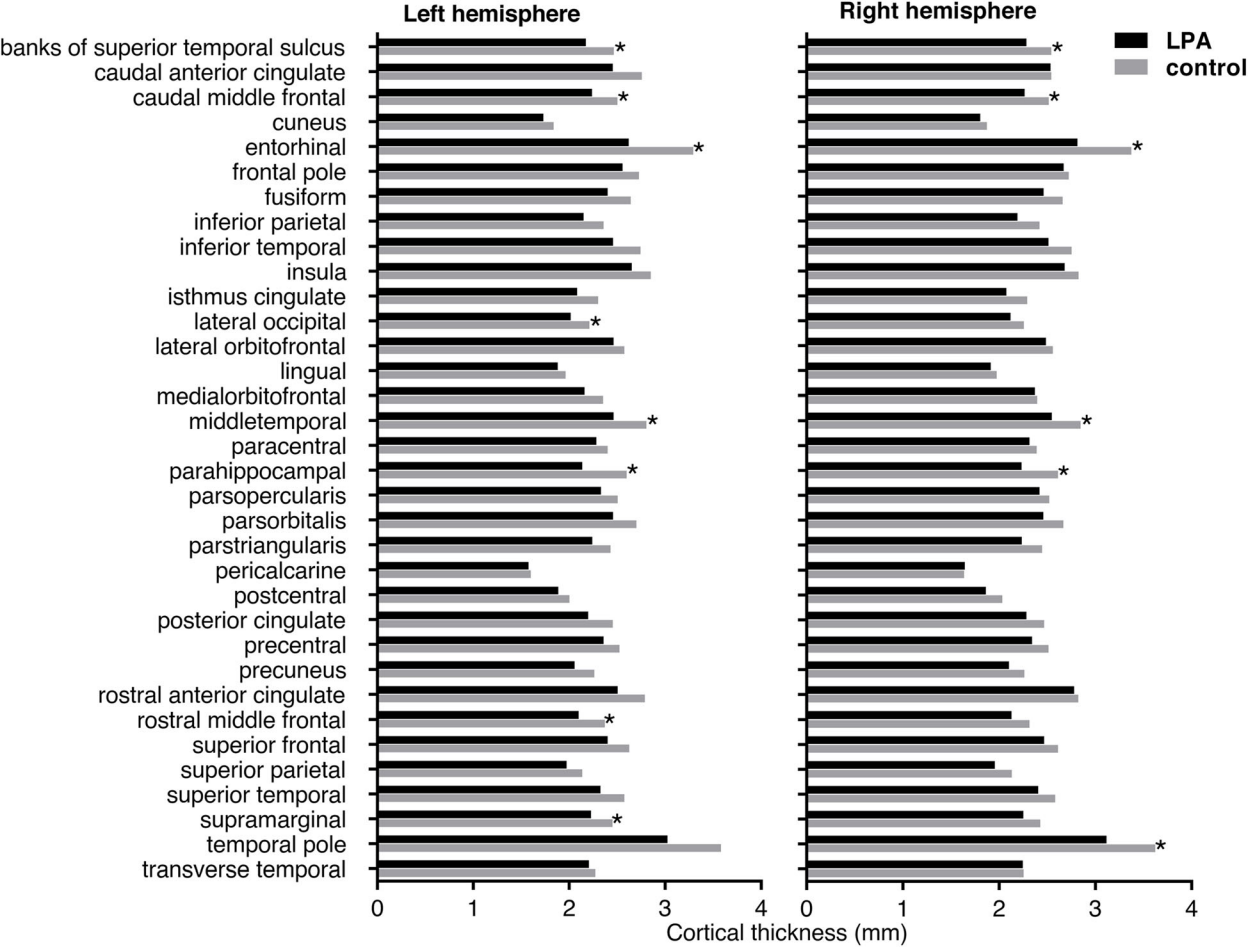
Cortical thickness of each cortical region in LPA and control groups. *Using GLM controlling for age, sex and total brain volume in *SPSS software*, LPA group was shown to have significant cortical thinning on the left cerebral hemisphere in the banks of the superior temporal sulcus (BSSTS) (*p* = 0.001), caudal middle frontal gyrus (CMFG) (*p* = 0.006), entorhinal cortex (EC) (*p* = 0.001), lateral occipital gyrus (LOG) (*p* = 0.005), middle temporal gyrus (MTG) (*p* = 0.002), parahippocampal gyrus (PHG) (*p* = 0.008), rostral middle frontal gyrus (RMFG) (*p* = 0.001) and supramarginal gyrus (SG) (*p* = 0.006), while on the right hemisphere in the BSSTS (*p* = 0.006), CMFG (*p* = 0.005), EC (*p* = 0.002), MTG (*p* = 0.005), PHG (*p* = 0.002) and temporal pole (TP) (*p* = 0.005)

The between group comparisons of the average thickness in each parcellated cortical area is shown in Fig. 1. On the left hemisphere, LPA group had significant cortical thinning in the banks of the superior temporal sulcus (BSSTS) (*p* = 0.001), caudal middle frontal gyrus (CMFG) (*p* = 0.006), entorhinal cortex (EC) (*p* = 0.001), lateral occipital gyrus (LOG) (*p* = 0.005), MTG (*p* = 0.002), parahippocampal gyrus (PHG) (*p* = 0.008), rostral middle frontal gyrus (RMFG) (*p* = 0.001) and supramarginal gyrus (SG) (*p* = 0.006). On the right hemisphere, LPA group had significant cortical thinning at the BSSTS (*p* = 0.006), CMFG (*p* = 0.005), EC (*p* = 0.002), MTG (*p* = 0.005), PHG (*p* = 0.002) and temporal pole (TP) (*p* = 0.005). On the other hand, we did not detect any region with significantly thinner cortex in the control group.

### Comparing cortical thickness between groups using vertex-by-vertex GLM analysis in QDEC

**Fig. 2:**
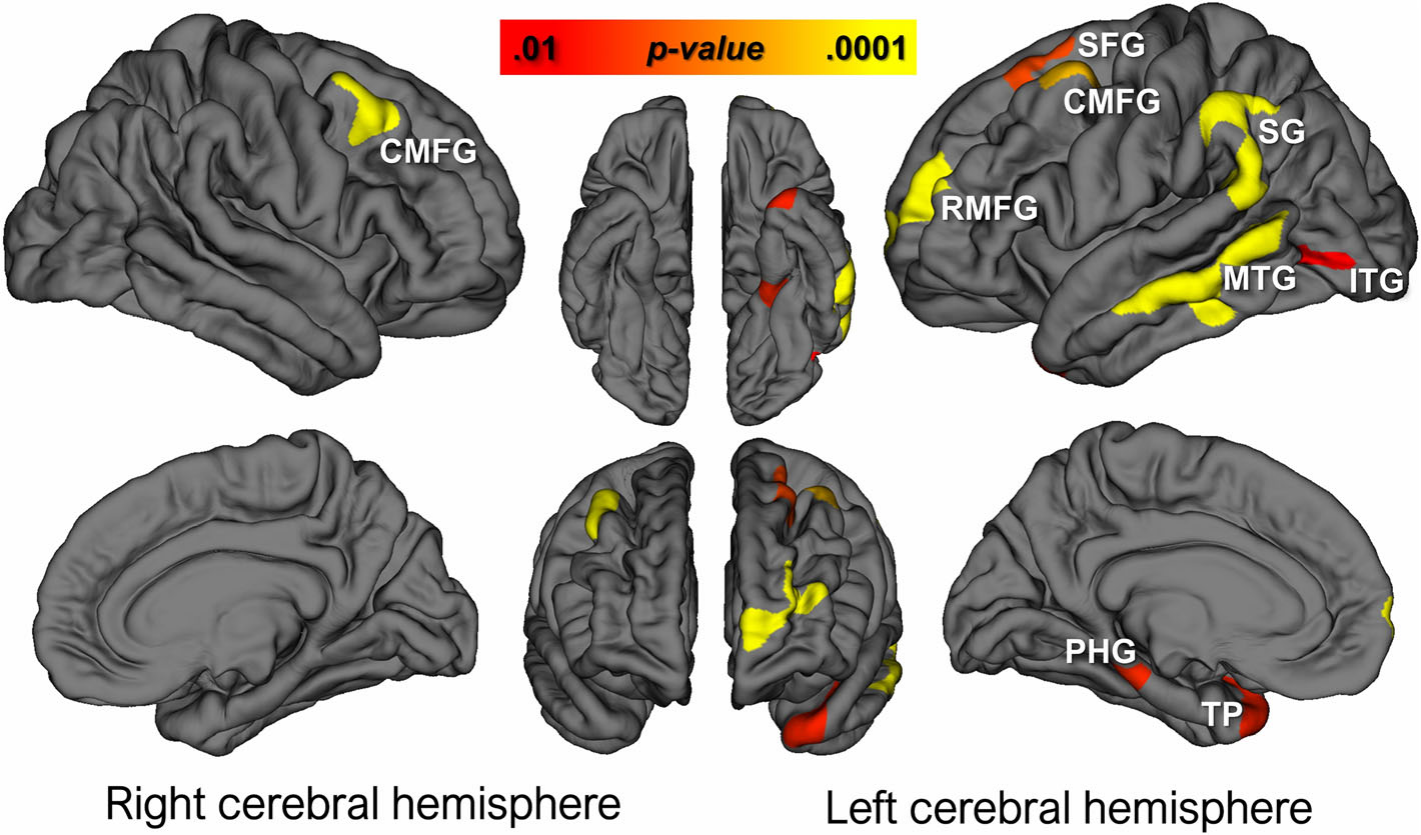
Comparing cortical thickness between groups using vertex-by-vertex GLM analysis in *Freesurfer’s QDEC interface.* Clusters showing significant cortical thinning in the LPA than in control groups are demonstrated on the cortical surface templates. On the left hemisphere, these clusters locate in the middle temporal gyrus (MTG) (area 1117.81 mm^2^, *p* = 0.0001), supramarginal gyrus (SG) (area 932.98 mm^2^, *p* = 0.0001), rostral middle frontal gyrus (RMFG) (area 791.38 mm^2^, *p* = 0.0001), caudal middle frontal gyrus (CMFG) (area 435.78 mm^2^, *p* = 0.0003), superior frontal gyrus (SFG) (area 334.68 mm^2^, *p* = 0.003), parahippocampal gyrus (PHG) (area 296.81 mm^2^, *p* = 0.005), temporal pole (TP) (area 290.13 mm^2^, *p* = 0.005), and inferior temporal gyrus (ITG) (area 264.06 mm^2^, *p* = 0.01). On the right hemisphere, the cluster locates in the CMFG (area 503.43 mm^2^, *p* = 0.0001). There was no cluster showing significantly thinner cortex in the control than in LPA groups

Clusters showing significant difference in cortical thickness between the two groups are demonstrated on the cortical maps of left and right cerebral hemispheres (Fig. 2). On the left hemisphere, the analysis showed many clusters that had thinner cortex in the LPA group. These located in the MTG (area 1117.81 mm^2^, *p* = 0.0001), SG (area 932.98 mm^2^, *p* = 0.0001), RMFG (area 791.38 mm^2^, *p* = 0.0001), CMFG (area 435.78 mm^2^, *p* = 0.0003), superior frontal gyrus (SFG) (area 334.68 mm^2^, *p* = 0.003), PHG (area 296.81 mm^2^, *p* = 0.005), TP (area 290.13 mm^2^, *p* = 0.005), and inferior temporal gyrus (ITG) (area 264.06 mm^2^, *p* = 0.01). Such cluster on the right hemisphere locate only in the CMFG (area 503.43 mm^2^, *p* = 0.0001). There was no cluster showing significantly thinner cortex in the control group.

## Discussion

This study investigated thinning pattern along the entire brain cortical surface in LPA patients. The results showed that LPA group had overall perspective of pronounced cortical thinning in more areas on the left than right hemisphere compared to control group, especially in the area around left TPJ including MTG, SG, ITG and LOC. This finding is consistent with the previous ones, which demonstrated that the area of peak cortical atrophy in LPA patients extended on the left temporal cortex and TPJ, while the temporal pole and medial temporal cortex were spared [6–9].

Apart from that typical imaging feature, we demonstrated the whole-hemispheric overall cortical thickness to be lower in LPA than in control group, regardless of the younger age and the slightly greater brain volume. Most parcellated cortical areas also tended to be thinner in LPA group (Fig. 1). In addition, the TMSE score was shown to have significant positive correlations with the whole-hemispheric overall cortical thickness. This may reflect the nature of neurodegenerative disorder which every single neuron is vulnerable. Yet, the mechanism how LPA patients had a distinct character of peak atrophy in the language area of the dominant hemisphere, in spite of common neuropathological substrate as AD, remains unresolved [3].

Interestingly, our analyses revealed that bilateral middle frontal gyri (MFG) and left SFG are significantly thinner in LPA patients comparing to control subjects. These clusters in the frontal regions were considerably large, especially on the left hemisphere which the clusters placed in both caudal and rostral parts of MFG. To our knowledge, frontal lobe involvement has been rarely described causing disorders in LPA patients. This finding, however, is quite consistent with some previous studies of cortical thickness in LPA patients reported the relative cortical thinning in the left frontal region [8, 19]. Regarding the language function of this frontal region, there was a study correlated the cortical thickness to language processing denoted that MFG and speech fluency were related [11]. This study also remarked that reduction of fluency such as impaired utterance and disrupted chain of spoken or written language can be seen in many language or speech disorders including non-fluent/agrammatic variant of PPA (nfvPPA, PPA-G), apraxia of speech and even LPA. Additionally, there was a report of some LPA patients presenting with word-finding difficulty and poor verbal fluency despite nearly normal naming ability [3]. Therefore, the involvement of MFG, the region responsible for verbal fluency, may alternatively underlie the classical symptom in some clinically diagnosed LPA patients. In other words, our analyses indicated that the classical symptoms of LPA, including failures in word retrieval and sentence repetition, are underlaid by abnormalities in the TPJ area or alternatively in the MFG. This finding may emphasize the complexity of neural substrate in association with language disorder, as a defect in the different steps of language processing potentially leads to an apparently identical phenotype of LPA. This inference implicates clinical practice as it reminds that an absolute decision on anatomical localization from a single clinical presentation of language disorder is not necessarily correct. For the LPA patients, further explorations regarding disease progression, prognosis, response to treatment, and even neuropathology in association with pattern of cortical thinning would have clinical benefits as well.

To discuss further, many Thai noun words are “noun phrases”, which are composed of several simple words [20]. For example, the word “driver” in Thai language is literally expressed as “man-drive-car”. Ordering of words in the noun phrase must also be in a correct sequence. As MFG is involved in sequential word ordering process [21], the thinning of MFG is thus possibly an exceptional feature in Thai LPA patients.

Other significant thinner regions and clusters in LPA group located in bilateral PHG, ECs and TPs. Involvement of the PHG and ECs may reflect the underlying AD neuropathology in majority of LPA patients [22]. As for TPs involvement, prominent atrophy of the left anterior temporal lobe and left TP has been known as a common pattern in semantic variant of PPA (svPPA, PPA-S) [7]. Although to a lesser extent, right anterior temporal region was also reported to be consistently atrophied in svPPA patients [23]. Thus, our finding regarding the thinning of TPs reflects that LPA manifestation possibly represents a prodromal stage of svPPA in some patients. This hypothesis is in accordance with the natural history of PPA syndrome, which the distinctive features of each PPA variant at early stage usually loss their uniqueness as the disease progresses to late stage, i.e. the progression trajectory [3, 24, 25].

Our finding about the alternative area of prominent cortical thinning in LPA patients is considered to be a strength in this study. The main limitation is the diagnosis of LPA, which was based on clinical diagnostic criteria combined with information from structural brain MRI. The use of functional imaging and biomarkers would possibly lead to the more accurate diagnosis. A between-group comparison by functional brain imaging technique, additional to structural MRI, would also strengthen the study result. These were unfortunately limited due to resource constraints. Besides, the present study lacks the information regarding formal linguistic tests in LPA patients as the Thai version of such tests have not been validated and primary language of all the patients is Thai. The TMSE, which had been validated and was used in our study, may not be the best assessment tool for patients with linguistic problem. Because good language capacity is required to complete tasks in several sections of the TMSE, i.e. not specific to the language section [26]. This was reflected in our results, which showed that LPA patients had significantly lower performance in both language and non-language sections of the TMSE.

Regarding the demographic data, proportion of male patient and age of the participants were markedly higher in LPA group. These occurred due to limitations in our resources, i.e. we were not able perform brain MRI in perfectly healthy and well-matched control individuals. The difference in proportion of sex, however, should not seriously confound our result since cortical areas known to be thicker in woman than in man locate in parietal and temporal regions of the right cerebral hemisphere [27]. Regarding the difference in age of participants, one may argue this potentially confounds the results since brain atrophy relates to aging process [28]. However, it is unlikely that this difference would significantly change our results as we demonstrated that the brain volume of the two groups were comparable. In fact, this difference in age should bias our result into the opposite direction because the control group was the one that were significantly older. In addition, we demonstrated that age and sex had no significant relationship to overall cortical thickness in our participants. Lastly, the present work is a single-centered study containing small numbers of LPA patients as we had to strictly follow the diagnostic criteria to avoid misdiagnosis. Also, there is a substantial difference in the number of participants between the groups. We recruited more participants in the control group in order to increase reliability of our analyses.

## Conclusion

This is an MRI study of LPA patients in Thailand, comparing the cortical thickness between LPA patients and control subjects. Besides the well-cognized left TPJ, we demonstrated that middle frontal gyri were significantly thinner in LPA patients, which indicates that poor verbal fluency may be an alternative underlying mechanism of language manifestations in some clinically diagnosed LPA patients. This reflects the multi-step nature of language processing in the brain, which defects in the different step of the process can lead to a single clinical phenotype of language dysfunction. We also observed substantial involvements of PHG, EC and TP in LPA group, which may associate with underlying Alzheimer’s disease pathology and progression trajectory nature of LPA.

## Supplementary Information

**Additional file 1: Supplementary Table 1** Correlation coefficients and *p*-values of the relationship analyses between TMSE score (language section score and total score) and thickness of each parcellated cortical region.

## Data Availability

The datasets used and/or analysed during the current study are available from the corresponding author on reasonable request.

## Abbreviations

AD: Alzheimer’s disease
BSSTS: Banks of superior temporal sulcus
CMFG: Caudal middle frontal gyrus
EC: Entorhinal cortex
FLAIR: Fluid-attenuated inversion recovery
GLM: General linear model
ITG: Inferior temporal gyrus
LOG: Lateral occipital gyrus
LPA: Logopenic progressive aphasia
lvPPA: Logopenic variant of primary progressive aphasia
MFG: Middle frontal gyrus
MRI: Magnetic resonance imaging
MTG: Middle temporal gyrus
nfvPPA: Non-fluent/agrammatic variant of primary progressive aphasia
RMFG: Rostral middle frontal gyrus
SFG: Superior frontal gyrus
SG: Supramarginal gyrus
STG: Superior temporal gyrus
svPPA: Semantic variant of primary progressive aphasia
TE: Echo time
TMSE: Thai Mental State Examination
TP: Temporal pole
TPJ: Temporo-parietal junction
TR: Relaxation time
