# Supplementary material for: Pattern of cortical thinning in logopenic progressive aphasia patients in Thailand
Source: BMC Neurol. 2021 Jan 13;21:22. doi: 10.1186/s12883-020-02039-x (PMC7805202; doi:10.1186/s12883-020-02039-x)
Supplement: Supplementary file 1 — Additional file 1: Supplementary Table 1 Correlation coefficients and p-values of the relationship analyses between TMSE score (language section score and total score) and thickness of each parcellated cortical region. [file 12883_2020_2039_MOESM1_ESM.pdf]

Supplementary Table 1 Correlation coefficients and p-values of the relationship analyses between TMSE score (language section score and total score) and thickness of each parcellated cortical region

		TMSE score (language section)	TMSE score (total)
**Left hemispheric banks of superior temporal sulcus thickness**	Correlation coefficient	.470	.551
	p-value	.009	.002
**Left hemispheric caudal anterior cingulate thickness**	Correlation coefficient	.513	.541
	p-value	.004	.002
**Left hemispheric caudal middle frontal thickness**	Correlation coefficient	.604	.741
	p-value	.000	.000
**Left hemispheric cuneus thickness**	Correlation coefficient	.281	.385
	p-value	.133	.036
**Left hemispheric entorhinal thickness**	Correlation coefficient	.465	.532
	p-value	.010	.002
**Left hemispheric fusiform thickness**	Correlation coefficient	.323	.454
	p-value	.082	.012
**Left hemispheric inferior parietal thickness**	Correlation coefficient	.485	.573
	p-value	.007	.001
**Left hemispheric inferior temporal thickness**	Correlation coefficient	.433	.546
	p-value	.017	.002
**Left hemispheric isthmus cingulate thickness**	Correlation coefficient	.410	.460
	p-value	.025	.011
**Left hemispheric lateral occipital thickness**	Correlation coefficient	.491	.547
	p-value	.006	.002
**Left hemispheric lateral orbitofrontal thickness**	Correlation coefficient	.289	.438
	p-value	.121	.016
**Left hemispheric lingual thickness**	Correlation coefficient	.166	.277
	p-value	.379	.138
**Left hemispheric medial orbitofrontal thickness**	Correlation coefficient	.499	.590
	p-value	.005	.001
**Left hemispheric middle temporal thickness**	Correlation coefficient	.594	.677
	p-value	.001	.000
**Left hemispheric parahippocampal thickness**	Correlation coefficient	.475	.591
	p-value	.008	.001
**Left hemispheric paracentral thickness**	Correlation coefficient	.253	.378
	p-value	.177	.039
**Left hemispheric pars opercularis thickness**	Correlation coefficient	.472	.581
	p-value	.008	.001
**Left hemispheric pars orbitalis thickness**	Correlation coefficient	.512	.606
	p-value	.004	.000
**Left hemispheric pars triangularis thickness**	Correlation coefficient	.564	.647
	p-value	.001	.000
**Left hemispheric pericalcarine thickness**	Correlation coefficient	.044	.127
	p-value	.819	.505
**Left hemispheric postcentral thickness**	Correlation coefficient	.475	.483
	p-value	.008	.007

*Highlighted cells indicate statistically significant analysis results

Supplementary Table 1 Correlation coefficients and p-values of the relationship analyses between TMSE score (language section score and total score) and thickness of each parcellated cortical region

**Left hemispheric posterior cingulate thickness**	Correlation coefficient	.457	.562
	p-value	.011	.001
**Left hemispheric precentral thickness**	Correlation coefficient	.478	.560
	p-value	.008	.001
**Left hemispheric precuneus thickness**	Correlation coefficient	.484	.590
	p-value	.007	.001
**Left hemispheric rostral anterior cingulate thickness**	Correlation coefficient	.480	.573
	p-value	.007	.001
**Left hemispheric rostral middle frontal thickness**	Correlation coefficient	.661	.774
	p-value	.000	.000
**Left hemispheric superior frontal thickness**	Correlation coefficient	.556	.681
	p-value	.001	.000
**Left hemispheric superior parietal thickness**	Correlation coefficient	.512	.603
	p-value	.004	.000
**Left hemispheric superior temporal thickness**	Correlation coefficient	.435	.502
	p-value	.016	.005
**Left hemispheric supramarginal thickness**	Correlation coefficient	.594	.684
	p-value	.001	.000
**Left hemispheric frontal pole thickness**	Correlation coefficient	.424	.365
	p-value	.020	.047
**Left hemispheric temporal pole thickness**	Correlation coefficient	.410	.505
	p-value	.024	.004
**Left hemispheric transverse temporal thickness**	Correlation coefficient	.228	.208
	p-value	.225	.270
**Left hemispheric insula thickness**	Correlation coefficient	.302	.375
	p-value	.105	.041
**Left hemispheric overall thickness**	Correlation coefficient	.582	.696
	p-value	.001	.000
**Right hemispheric banks of superior temporal sulcus thickness**	Correlation coefficient	.417	.556
	p-value	.022	.001
**Right hemispheric caudal anterior cingulate thickness**	Correlation coefficient	.064	.165
	p-value	.737	.383
**Right hemispheric caudal middle frontal thickness**	Correlation coefficient	.593	.689
	p-value	.001	.000
**Right hemispheric cuneus thickness**	Correlation coefficient	.249	.322
	p-value	.184	.083
**Right hemispheric entorhinal thickness**	Correlation coefficient	.369	.424
	p-value	.045	.020
**Right hemispheric fusiform thickness**	Correlation coefficient	.275	.381
	p-value	.141	.038
**Right hemispheric inferior parietal thickness**	Correlation coefficient	.483	.596
	p-value	.007	.001
**Right hemispheric inferior temporal thickness**	Correlation coefficient	.428	.550
	p-value	.018	.002

*Highlighted cells indicate statistically significant analysis results

Supplementary Table 1 Correlation coefficients and p-values of the relationship analyses between TMSE score (language section score and total score) and thickness of each parcellated cortical region

**Right hemispheric isthmus cingulate thickness**	Correlation coefficient	.421	.467
	p-value	.021	.009
**Right hemispheric lateral occipital thickness**	Correlation coefficient	.291	.372
	p-value	.119	.043
**Right hemispheric lateral orbitofrontal thickness**	Correlation coefficient	.242	.355
	p-value	.197	.054
**Right hemispheric lingual thickness**	Correlation coefficient	.168	.226
	p-value	.373	.230
**Right hemispheric medial orbitofrontal thickness**	Correlation coefficient	.119	.182
	p-value	.533	.335
**Right hemispheric middle temporal thickness**	Correlation coefficient	.476	.581
	p-value	.008	.001
**Right hemispheric parahippocampal thickness**	Correlation coefficient	.494	.595
	p-value	.006	.001
**Right hemispheric paracentral thickness**	Correlation coefficient	.215	.351
	p-value	.253	.057
**Right hemispheric pars opercularis thickness**	Correlation coefficient	.244	.327
	p-value	.194	.077
**Right hemispheric pars orbitalis thickness**	Correlation coefficient	.413	.473
	p-value	.023	.008
**Right hemispheric pars triangularis thickness**	Correlation coefficient	.586	.668
	p-value	.001	.000
**Right hemispheric pericalcarine thickness**	Correlation coefficient	.024	.044
	p-value	.902	.818
**Right hemispheric postcentral thickness**	Correlation coefficient	.430	.460
	p-value	.018	.010
**Right hemispheric posterior cingulate thickness**	Correlation coefficient	.405	.526
	p-value	.027	.003
**Right hemispheric precentral thickness**	Correlation coefficient	.461	.478
	p-value	.010	.007
**Right hemispheric precuneus thickness**	Correlation coefficient	.474	.547
	p-value	.008	.002
**Right hemispheric rostral anterior cingulate thickness**	Correlation coefficient	.091	.237
	p-value	.634	.207
**Right hemispheric rostral middle frontal thickness**	Correlation coefficient	.540	.647
	p-value	.002	.000
**Right hemispheric superior frontal thickness**	Correlation coefficient	.456	.547
	p-value	.011	.002
**Right hemispheric superior parietal thickness**	Correlation coefficient	.518	.611
	p-value	.003	.000
**Right hemispheric superior temporal thickness**	Correlation coefficient	.244	.307
	p-value	.195	.099
**Right hemispheric supramarginal thickness**	Correlation coefficient	.435	.485
	p-value	.016	.007

*Highlighted cells indicate statistically significant analysis results

Supplementary Table 1 Correlation coefficients and p-values of the relationship analyses between TMSE score (language section score and total score) and thickness of each parcellated cortical region

**Right hemispheric frontal pole thickness**	Correlation coefficient	.183	.212
	p-value	.332	.261
**Right hemispheric temporal pole thickness**	Correlation coefficient	.355	.350
	p-value	.055	.058
**Right hemispheric transvers temporal thickness**	Correlation coefficient	.051	.014
	p-value	.790	.942
**Right hemispheric insula thickness**	Correlation coefficient	.172	.270
	p-value	.364	.150
**Right hemispheric overall thickness**	Correlation coefficient	.513	.615
	p-value	.004	.000

*Highlighted cells indicate statistically significant analysis results
